# Complex interplay between intrinsic and extrinsic drivers of long-term survival trends in southern elephant seals

**DOI:** 10.1186/1472-6785-7-3

**Published:** 2007-03-27

**Authors:** Siobhan C de Little, Corey JA Bradshaw, Clive R McMahon, Mark A Hindell

**Affiliations:** 1 School for Environmental Research, Charles Darwin University, Darwin, Northern Territory 0909, Australia; 2 Antarctic Wildlife Research Unit, School of Zoology, University of Tasmania, Private Bag 05, Hobart, Tasmania 7001, Australia

## Abstract

**Background:**

Determining the relative contribution of intrinsic and extrinsic factors to fluctuations in population size, trends and demographic composition is analytically complex. It is often only possible to examine the combined effects of these factors through measurements made over long periods, spanning an array of population densities or levels of food availability. Using age-structured mark-recapture models and datasets spanning five decades (1950–1999), and two periods of differing relative population density, we estimated age-specific probabilities of survival and examined the combined effects of population density and environmental conditions on juvenile survival of southern elephant seals at Macquarie Island.

**Results:**

First-year survival decreased with density during the period of highest population size, and survival increased during years when the Southern Oscillation Index (SOI) anomaly (deviation from a 50-year mean) during the mother's previous foraging trip to sea was positive (i.e., El Niño). However, when environmental stochasticity and density were considered together, the effect of density on first-year survival effectively disappeared. Ignoring density effects also leads to models placing too much emphasis on the environmental conditions prevailing during the naïve pup's first year at sea.

**Conclusion:**

Our analyses revealed that both the state of the environment and population density combine to modify juvenile survival, but that the degree to which these processes contributed to the variation observed was interactive and complex. This underlines the importance of evaluating the relative contribution of both the intrinsic and extrinsic factors that regulate animal populations because false conclusions regarding the importance of population regulation may be reached if they are examined in isolation.

## Background

A central aim in population biology is to discern the relative contribution of intrinsic (density-regulated) and extrinsic (environmental) factors to fluctuations in population size and demographic composition, with increasing emphasis placed on quantifying the complex interplay between the two [1-4]. The mounting number of long-term ecological studies available for the measurement of population dynamical parameters, although still relatively rare, is providing a more refined understanding of the combined effects of these mechanisms [4-8]. For instance, investigating the relationships between population density, environmental conditions and survival probability using mark-recapture techniques has provided important advances in this regard [e.g., [9-11]].

Given that populations of large, long-lived mammals tend to have a relatively low capacity for growth due to their long generation times and low reproductive output [12], it is hypothesized that intrinsic factors should regulate growth only near carrying capacity. Indeed, there is good evidence that this is the case in many large mammal species [8,12,13], with many studies concluding that extrinsic factors are the predominate drivers of change when populations are below carrying capacity [12,14,15]. However, the complex relationships that exist between extrinsic and intrinsic control mean that there is no species for which there is a complete understanding of how abundance is regulated over the complete range of population densities [16]. Another bugbear is that many populations with a high degree of age-dependent fecundity and mortality may not reveal density dependence if the time series used in the investigation is short relative to generation time [4,12,17]. In practice, it is usually only possible to examine the combined effects of density and environmental conditions through measurements made over long periods spanning an array of population densities or levels of food availability. As such, there are only a few case studies where this has been done for long-lived mammals, and most of those have focussed on island populations of ungulates [2,18,19].

Changes in the population size of large marine predators is potentially indicative of larger ecosystem changes given that their predominate regulator appears to be environmental stochasticity influencing food availability over vast oceanic foraging regions (e.g., [20-22]). Upper trophic-level marine predators such as seabirds and seals are particularly amenable to the examination of such mechanistic hypotheses because they are easily monitored during their obligatory onshore breeding phase [21]. Access to such rare datasets is particularly important given the predictions of climate change over the next few decades [22,23], and recent evidence for broad-scale changes in population trends in birds and mammals throughout, for example, the Southern Ocean [24-29].

**Figure 1:**
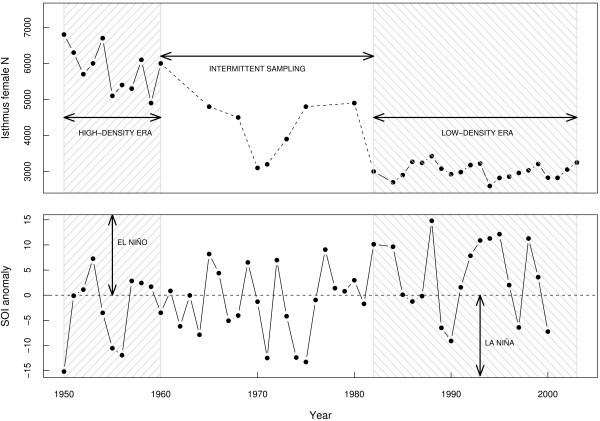
**Macquarie Island southern elephant seal abundance trends (1951 – 2003) and the Southern Oscillation Index over that period**. (Top panel) Abundance trends of the isthmus population of breeding females at Macquarie Island from 1951 to 2003. Two main census periods emerge (1) between 1951 and 1960 (the relatively high-density era) and (2) from 1993 to 1999 (the low-density era). (B) El Niño-Southern Oscillation (ENSO) conditions as measured by the Southern Oscillation Index (SOI) anomaly over the elephant seal foraging period from January to October between 1950 and 2001. High positive values of the SOI anomaly indicate El Niño conditions, and high negative values indicate La Niña conditions.

The well-documented population decline and possible recent stabilization of one of the most wide-ranging Southern Ocean predators, the southern elephant seal (*Mirounga leonina*) at Macquarie Island, has been the focus of intensive demographic studies for over fifty years [22,26,30]. Population censuses from the 1940s to the present and capture-mark-recapture studies from the 1950s and 1990s have provided extensive demographic data for this population at both low and high population densities [22,26,30-32]. There is strong evidence that this population responds to environmental stochasticity via modifications to individual survival given that this parameter is highly sensitive to the at-sea foraging conditions experienced by an individual over its predominately aquatic life cycle [22,32]. Foraging elephant seals breeding at Macquarie Island range widely over millions of square kilometres of the Southern Ocean [21,33,34], and it has been established that their feeding areas are associated to some extent with the pack ice zone and colder sea surface temperatures – these environmental conditions are known to fluctuate with El Niño-Southern Oscillation (ENSO) patterns [22,32,33]. In this region, ENSO follows an approximate seven- to eight-year cycle during which time ocean productivity can fluctuate substantially [35,36] (Fig. 1B).

It has been suggested that changes in ocean conditions affect southern elephant seals either directly by modifying the availability of food resources, or indirectly by affecting sea ice dynamics and hence, ocean productivity [22]. While some studies have shown that survival [37], weaning mass [38] and weanling sex ratio [39] are reduced or modified during El Niño conditions, the Macquarie Island population of elephant seals has shown a consistent positive relationship between El Niño and pup survival [40]. These contrasting relationships may arise from the different climatic conditions associated with ENSO events in different regions of the Southern Ocean [41].

There is also some evidence for density regulation in southern elephant seal populations, mainly via space limitation on land while breeding [42-44]. However, competition for food at high population densities may also occur during the at-sea foraging phase [22,45-47]. In this paper we expand greatly on previous work by amalgamating capture-mark-recapture data collected over two extended periods of differing population density at Macquarie Island: (1) the years between 1951 and 1960 when the population was relatively abundant, and (2) between 1993 and 1999 when it was approximately 50 % smaller. Our main aim was to identify whether there is evidence for density and environmental effects on survival rates and how these mechanisms combine to explain the observed phenomenological trends of population size over the last 50 years. We achieve this by (1) assessing the concurrent age-, and sex- specific survival at the two different density levels, (2) testing for density dependence in adult and first-year survival between and at both densities, and (3) testing for the effects of environmental variation as represented by ENSO on adult and first-year survival.

## Results

### Phenomenological evidence for density dependence

**Figure 2:**
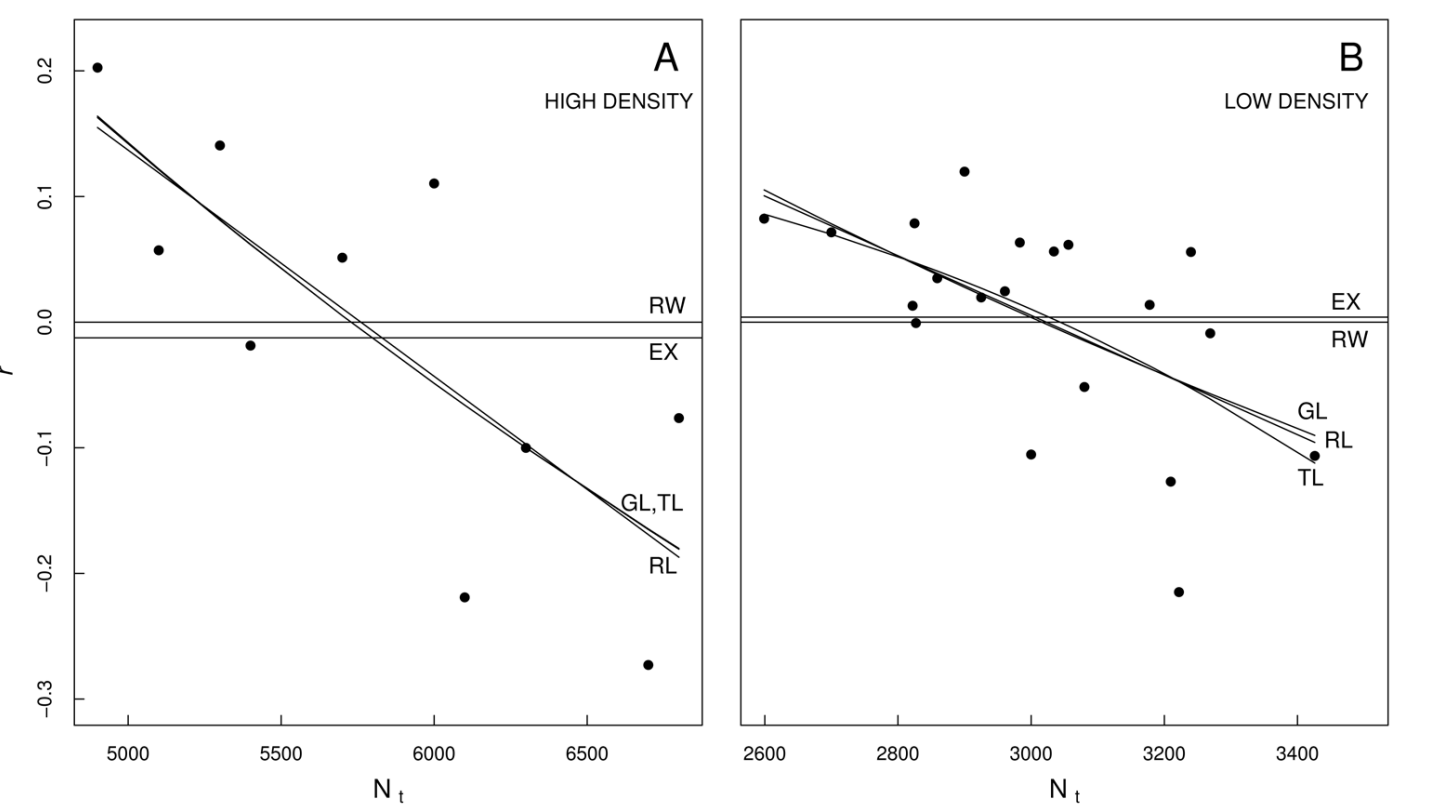
**Rate of population change versus abundance**. Intrinsic rate of population change (*r *= log [N_t+1_/N_t_]) versus N_t _(abundance) for the breeding female southern elephant seal population at the Macquarie Island isthmus during (A) the high-density era (1951–1960) and (B) the low-density era (1993–1999). Five population dynamics models (RW = random walk, EX = exponential growth, RL = Ricker-logisitc growth, GL = Gompertz-logistic growth and TL = *θ*-logistic growth; see Methods) were fitted to the relationship of *r *versus N_t_. The sum of the Akaike Information Criterion (corrected for small sample sizes – AIC_*c*_) weights over the three density-dependent models considered (RL, GL and TL) show 77.4 % strength of evidence for density dependence during the high-density era (A) and 89.3 % support for the phenomenon during the low-density era (B).

For the high-density time series (1951–1965), there was good evidence for density-dependent population growth, with the summed Akaike's Information Criterion corrected for small samples size (AIC_*c*_) weights for the three density-dependent models = 77.4 % (Fig. 2A, Table 1). There was also strong evidence for density-dependent population growth for the low-density time series (1993–1999), where the summed AIC_*c *_weight for the density-dependent models was 89.3 % (Fig. 2B, Table 1).

**Table 1:** Evidence for density dependence using phenomenological time series data

		***w*AIC**_*c*_					**%DD**
**Time series era**	***q***	**RW**	**EX**	**RL**	**GL**	**TL**	**Σ*w*AIC_*c*_**
High-density (1951–1964)	10	0.187	0.039	0.372	0.383	0.019	77.4
Low-density (1993–1999)	21	0.081	0.025	0.412	0.380	0.101	89.3

Sample-size corrected Akaike weights (*w*AIC_*c*_) and the number of yearly transitions (*q*) for density-independent models: random walk (RW), and exponential (EX), and density-dependent models: Ricker-logisitc (RL), Gompertz logistic (GL), and *θ*-logisitc (TL), of the population growth of southern elephant seals during the high-density era and low-density eras. The sum of the AIC_*c *_weights for the density-dependent models represents the combined percentage support for density dependence (%DD).

### Capture-mark-recapture

The parametric goodness-of-fit bootstrap results for both the high- and low-density eras showed evidence for lack of fit to the Cormack-Jolly-Seber (CJS) model assumptions (*P *< 0.0001) and over-dispersion. Non-compliance with the CJS assumptions, in particular the assumption that all animals from both datasets had the same resighting probability, may have influenced survival estimates. However, it has been shown that increased variation in resighting rate inflates the variance of survival estimates rather than their means [48].

To account for over-dispersion, the inflation factor, c, was used to correct the AIC values in all remaining analyses. For the high-density dataset, *ĉ *was calculated for each model set using the observed and simulated deviance and deviance degrees of freedom. For the low-density dataset, the values of *ĉ *calculated for each model set were considered too high (> 3) to be incorporated directly into the correction of AIC, so the model sets were compared with values of *ĉ *ranging from 1 to 10 to look for major discrepancies in first-year survival estimates and model weighting [see Additional file 1, tables 1 to 3].

During the low-density era and for the model set examining the effect of age, the model-averaged parameter estimates for first-year survival varied by a maximum of ± 2 % with *ĉ *ranging from 1 to 10 [see Additional file 1, table 1]. Likewise, model rankings only changed relative to the recapture probability *p *[see Additional file 1, table 1]. In the sex-effect model set, model-averaged parameter estimates for first-year survival varied by only 0.1 % with changes to *ĉ*, and the general model *ϕ *(age*time)*p*(time) had > 83 % of the model weight for all values of *ĉ *[see Additional file 1, table 1]. Varying *ĉ *within the SOI-covariate models [see Additional file 1, table 2A] and the density models [see Additional file 1, table 2B] also indicated little change to the model ranking with respect to survival probability, although there was some bias in the estimates of survival probability (see below). Models examining the effects of population density and SOI together demonstrated little bias (2 %) in parameter estimates with varying *ĉ*, although model rankings varied substantially [see Additional file 1, tables 3]. It should be noted, however, that the presence of excessive over-dispersion provides important information regarding the population structure in its own right. Previous work has determined that capture heterogeneity and survival vary as a function of weaning mass [49], so a certain degree of over-dispersion is expected.

### Sex and age effects

For the high-density era, the model that incorporated time- and age-based survival had over 99.9 % of the model weight (Table 2). Neither first-year or adult survival varied with gender (the general model, *ϕ *(age*time) *p*(time) was allocated > 99.9 % of the model weight; Table 3); therefore, male and females were pooled for all subsequent analyses. The most parsimonious model for the low-density era was one that incorporated time and age effects on survival (Table 2). There was no evidence that first-year survival varied with gender (the model ignoring the effect of gender accounted for > 99 % of the *w*QAIC_*c*_; Table 3), so males and females were again pooled for all subsequent analyses.

**Table 2:** Model ranking for models estimating age-specific survival and recapture probability

**Model**	**ΔQAIC**_*c*_	***w*QAIC**_*c*_	***k***
High density (1951–1964)			
*ϕ*(**age-t/t**) *p*(**age-t/t**)	0.000	0.948	38
*ϕ*(**age-t/t**) *p*(**t**)	5.820	0.052	29
*ϕ*(**t**) *p*(**age**-**t**/**t**)	19.140	<0.001	27
*ϕ*(**t**) *p*(**t**)	49.100	<0.001	22
*ϕ*(**.**) *p*(**t**)	122.780	<0.001	15
			
Low density (1993–1999)			
*ϕ*(**age**-**t**/**t**) *p*(**age**-**t**/**t**)	0.000	0.999	17
*ϕ*(**age**-**t**/**t**) *p*(**t**)	19.670	<0.001	13
*ϕ*(**t**) *p*(**age**-**t**/**t**)	72.570	<0.001	13
*ϕ*(**t**) *p*(**t**)	107.070	<0.001	9
*ϕ*(**.**) *p*(**t**)	112.770	<0.001	7

Effects of time (**t**), and age (**age-juvenile/adult**) on the probability of survival (*ϕ*) and recapture (*p*) of southern elephant seals during the high- (1951–1964) and low-density (1993–1999) eras. Models are ranked according to their Akaike weights (*w*QAIC_*c*_), the relative change in AIC_*c *_score (ΔQAIC_*c*_), and number of parameters (*k*) based on an inflation factor (*ĉ*) of 1.3664.

**Table 3:** Model ranking for models estimating age- and sex-specific survival and recapture probability

**Model**	**ΔQAIC**_*c*_	***w*QAIC**_*c*_	***k***
High density (1951–1964)			
*ϕ*(**age-t/t**) *p*(**t**)	0.000	0.999	25
*ϕ*(age-sex*t/t) *p*(**t**)	13.640	0.001	34
*ϕ*(**age**-sex*t/sex*t) *p*(**t**)	21.430	<0.001	40
*ϕ*(**t**) *p*(**t**)	125.470	<0.001	21
*ϕ*(sex*t) *p*(**t**)	137.080	<0.001	30
			
Low density (1993–1999)			
*ϕ*(**age**-**t**/**t**) *p*(**t**)	0.000	0.928	13
*ϕ*(**age**-sex*t/**t**) *p*(**t**)	5.690	0.054	17
*ϕ*(**age**-sex*t/sex*t) *p*(**t**)	7.890	0.018	22
*ϕ*(**t**) *p*(**t**)	125.440	<0.001	9
*ϕ*(sex*t) *p*(**t**)	126.950	<0.001	14

Effects of time (**t**), age (**age-juvenile/adult**), and sex (**sex**) on the probability of survival (*ϕ*) and recapture (*p*) in southern elephant seals during the high- (1951–1964) and low-density (1993–1999) eras. Models are ranked according to their Akaike weights (*w*QAIC_*c*_), the relative change in AIC_*c *_score (ΔQAIC_*c*_), and number of parameters (*k*) based on an inflation factor (*ĉ*) of 1.3559.

### Environmental conditions and density considered separately

**Figure 3:**
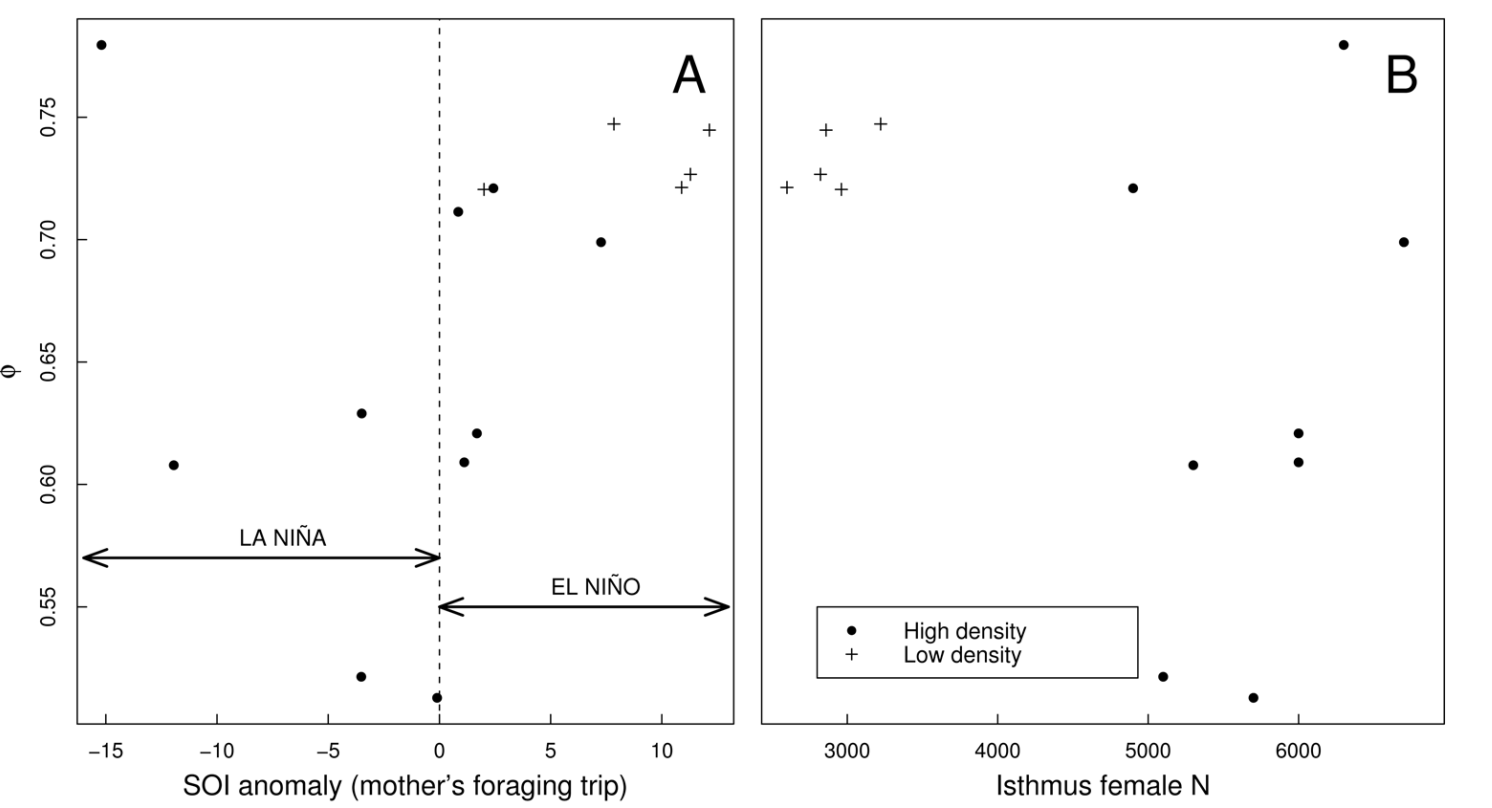
**Apparent survival probability of yearling southern elephant seals versus the Southern Oscillation Index and population size**. Model-averaged, time-variant estimates of mean apparent survival (*ϕ*) for yearling southern elephant seals at Macquarie Island plotted as a function of (A) the Southern Oscillation Index (SOI) anomaly over the mother's previous foraging trip (January to October) between 1950 and 2001 (high positive values of the SOI anomaly indicate El Niño conditions; high negative values indicate La Niña conditions), and (B) the number of breeding females counted on the isthmus of Macquarie Island that year.

For the high-density era, there was strong evidence that the probability of both first-year and adult survival varied with the standardized SOI (information-theoretic evidence ratio [*ER*] = 5881; Table 4). The most parsimonious model contained the SOI covariate representing the environmental conditions during the time naïve seals were foraging (*ER *= 4732, Table 4). For the low-density era and over all values of *ĉ*, > 74 % of the model weight was allocated to models with first-year survival varying with SOI measured during the pregnant mother's pre-partum foraging trip (Table 4; Fig. 3A). However, the model-averaged estimates of first-year survival varied by 63 % over the range of *ĉ *examined. Despite this variation in first-year survival, this model set clearly shows that environmental conditions (expressed as SOI) during the year of the mother's pre-partum foraging trip describe an important component of the variation in first-year survival (*ER *> 3.5).

**Table 4:** Model ranking for models estimating age-specific survival and recapture probability as a function of environmental stochasticity

**Model**	**ΔQAIC**_*c*_	***w*QAIC**_*c*_	***k***
High density (1951–1964)			
*ϕ*(**age**-pup*t/pup*t) *p*(**t**)	0.000	0.804	35
*ϕ*(**age**-mother*t/mother*t) *p*(**t**)	2.830	0.195	34
*ϕ*(**age**-**t**/**t**) *p*(**t**)	16.950	<0.001	30
*ϕ*(**age**-pup*t/**t**) *p*(**t**)	24.810	<0.001	34
*ϕ *(**age**-mother*t/**t**) *p*(**t**)	24.820	<0.001	34
			
Low density (1993–1999)			
*ϕ *(**age**-mother*t/mother*t)*p*(**t**)	0.000	0.767	24
*ϕ *(mother*t)*p*(**t**)	2.380	0.233	18
*ϕ *(**age**-pup*t/pup*t)*p*(**t**)	17.060	<0.001	22
*ϕ *(**age**-**t**/**t**)*p*(**t**)	32.800	<0.001	17
*ϕ *(**age**-pup*t/**t**)*p*(**t**)	40.820	<0.001	21

Effects of age (**age**-**juvenile/adult**), time (**t**), and environmental conditions represented by the Southern Oscillation Index (SOI) during the newly weaned seal's foraging period (Jan-Oct) (**pup**), and during a mother's pre-partum foraging period (Jan-Oct of the previous year) (**mother**) on the probability of survival (*ϕ*) of southern elephant seals during the high- (1951–1964) and low-density (1993–1999) eras. Models are ranked according to their Akaike weights (*w*QAIC_*c*_), the relative change in AIC_*c *_score (ΔQAIC_*c*_), and number of parameters (*k*) based on an inflation factor (*ĉ*) of 1.3861.

When the density covariate was considered alone, there was evidence that first-year survival varied with annual density in the high-density era (*ER *= 8.5; Table 5; Fig. 3B). For the low-density era, model weightings varied substantially over the range of *ĉ*; however, estimates of first-year survival varied by only 2 %. There was weak evidence for a density effect on first-year survival during this era (Table 5; Fig. 3B).

**Table 5:** Model ranking for models estimating age-specific survival and recapture probability as a function of population density

**Model**	**ΔQAIC**_*c*_	***w*QAIC**_*c*_	***k***
High density (1951–1964)			
*ϕ*(**age**-**density**/**t**) *p*(**t**)	0.000	0.894	14
*ϕ*(**age**-**t**/**t**) *p*(**t**)	4.270	0.106	18
*ϕ*(**t**) *p*(**t**)	14.860	0.001	13
*ϕ*(**age**-**dlag**/**t**) *p*(**t**)	16.960	<0.001	16
*ϕ*(**age**-**density**/**density**) *p*(**t**)	21.410	<0.001	14
			
Low density (1993–1999)			
*ϕ*(**age**-**density**/**t**) *p*(**t**)	0.000	0.399	10
*ϕ*(**age**-**t**/**t**) *p*(**t**)	0.720	0.278	13
*ϕ*(**age**-**dlag**/**t**) *p*(**t**)	0.960	0.246	10
*ϕ*(**age**-**density**/**density**) *p*(**t**)	3.310	0.076	10
*ϕ*(**age**-**dlag**/**dlag**) *p*(**t**)	17.940	<0.001	10

Effects of age (**age**-**juvenile/adult**), time (**t**), density of breeding females (**density**), and density of breeding females lagged by one year (**dlag**) on the probability of survival (*ϕ*) in southern elephant seals during the high- (1951–1964) and low-density (1993–1999) eras. Models are ranked according to their Akaike weights (*w*QAIC_*c*_), the relative change in AIC_*c *_score (ΔQAIC_*c*_), and number of parameters (*k*), based on an inflation factor (*ĉ*) of 1.7122.

### Combining intrinsic and extrinsic factors

During the high-density era, the model including the SOI values measured during the mother's pre-partum foraging trip had > 99 % of the model weight (Table 6, Fig. 3A), but there was no evidence that density explained additional variance in first-year survival (*ER *<< 1, Table 6). When population density was low, the high degree of variation in model weighting with changes to *ĉ *made determining the combined effects of these two covariates on first-year survival suspect (i.e., the variance among survival estimates was 175 %) [see Additional file 1, table 3]. Nonetheless, we assumed the same *ĉ *value from the high-density era to contrast models; this revealed that the models with the most support (combined QAIC weights > 99 %) incorporated the mother's foraging trip SOI, and there was little evidence for a density or pup-year SOI anomaly effect (Table 3).

**Table 6:** Model ranking for models estimating age-specific survival and recapture probability as a function of environmental stochasticity and population density

**Model**	**ΔQAIC**_*c*_	***w*QAIC**_*c*_	***k***
High density (1951–1964)			
*ϕ*(**age**-mother*t/mother*t) *p*(**t**)	0.000	0.995	19
*ϕ*(**age**-pup*t/pup*t) *p*(**t**)	11.300	0.004	24
*ϕ*(mother*t) *p*(**t**)	14.330	0.001	21
*ϕ*(pup*t) *p*(**t**)	16.130	<0.001	21
*ϕ*(**age**-**density**/**t**) *p*(**t**)	17.810	<0.001	15
			
Low density (1993–1999)			
*ϕ*(**age**-mother*t/mother*t) *p*(**t**)	0.000	0.551	22
*ϕ*(mother*t) *p*(**t**)	1.640	0.242	17
*ϕ*(**age**-**t**/mother*t) *p*(**t**)	1.970	0.206	23
*ϕ*(**age**-pup*t/pup*t) *p*(**t**)	13.050	0.001	20
*ϕ*(**age**-density+mother*t/density+mother*t) *p*(**t**)	14.200	<0.001	12

Effects of age (**age**-**juvenile/adult**), time (**t**), density of breeding females (**density**), density of breeding females lagged by one year (**dlag**) and environmental conditions (SOI during a newly weaned seal's foraging period [**pup**] and during a mother's pre-partum foraging period [**mother**] on the probability of survival (*ϕ*) of southern elephant seals during the high- (1951–1964) and low-density (1993–1999) eras. Models are ranked according to their Akaike weights (*w*QAIC_*c*_), the relative change in AIC_*c *_score (ΔQAIC_*c*_), and number of parameters (*k*), based on an inflation factor (*ĉ*) of 1.7122.

## Discussion

Determining the factors that regulate populations through time can be complex because even the simplest nonlinear, density-dependent population models may exhibit a large range of complex dynamic behaviours [50,51]. While environmental stochasticity tends to inflate the variance in population size and demographic rates, negative density feedback at high population sizes has the opposite effect [2,4]. However, density regulation may become undetectable at lower population sizes [13] or when environmental conditions are favourable [12,52]. Using an extensive dataset collected from a long-lived mammal, we found that first-year survival varied as predicted with population density, but only when population size was relatively high and when models ignored indices of environmental stochasticity.

When the effects of density and environmental variation were examined together, the negative density feedback mechanism was apparently overwhelmed by the more dominant influence of stochastic environmental forcing. This observation underscores the importance of examining the competing and complex interaction between environmental control and density regulation over a large range of population sizes, especially in long-lived species susceptible to high environmental stochasticity [53].

These results suggest that density regulation in this system may operate when populations are at or near carrying capacity – a state where intra- and inter-specific competition for resources and intra-specific competition for mates is likely to be highest. This notion is consistent with data from studies investigating the dynamics of other long-lived mammals [12,13]. However, the magnitude of these effects is dwarfed by density-independent stochastic environmental conditions that affect food availability during a pregnant mother's foraging trip. Indeed, this forcing also overshadowed any negative influences on survival experienced during a naïve seal's first trip to sea, despite previous, albeit weak, evidence that environmental conditions during that period influence first-year survival [22]. Although the phenomenological evidence for density dependence is pervasive across many different taxa [17], including the species under study, high environmental variation can sometimes mask even strong density dependence, especially if the effects are lagged [54-56]. Nonetheless, we found evidence for density regulation that would not have been detected using the low-density dataset alone, demonstrating the complex meshing of endogenous and exogenous forces in shaping animal population sizes [55].

Previous work has shown that many pinniped species demonstrate strong density dependence in various demographic rates and life history traits. [46,52,57-59], although these may be detectable only during poor-resource years [52]. Density dependence in elephant seals has been shown to operate mainly during breeding where concentrated adult aggregations onshore can directly affect pup survival or the age at first reproduction [43,44,46,60] even though the exact form and strength of density dependence acting in this species is still a matter of some debate [22,47]. There is also ample evidence that pinnipeds demonstrate density-regulated somatic growth rates, with lower growth experienced at high population densities [57,61].

The incorporation of density effects into the models considered also revealed the dominant mechanisms by which environment stochasticity controls population abundance patterns over time. When environmental stochasticity (expressed as the SOI) was examined without the effects of density, the most parsimonious model predicted that the conditions during an individual's first year of life best explained variation in survival when population size was high (Table 4). However, when the effects of density were also included in the models, there was more evidence that the environmental conditions experienced by the mother when she was gaining body reserves that would eventually sustain her pup were most important (Table 6). Had we failed to consider density effects directly, we would have erroneously concluded the mechanism by which population density exerts its influence on dampening environmentally induced variation in life history traits. With density included in the model set, the pregnant mother's environmental context clearly emerged as the most dominant force in shaping her offspring's survival probability. This supports previous work suggesting that wean mass, an indirect expression of the mother's capacity to sequester sufficient resources prior to giving birth, was the most important determinant of first-year survival [22]. However, unlike that previous study, our analyses add another piece to the puzzle by demonstrating the degree to which environmental stochasticity in the mother's foraging phase dominates intrinsic regulation.

We must also consider that the weak effects of population density on first-year survival are unlikely to capture the full mechanistic component of density regulation in this population, especially given the strong phenomenological evidence for density dependence in both relative-density periods. In addition to vital rates such as survival, density-dependent regulation may apply to other aspects of a species' biology, such as growth, behaviour, incidence of disease and distribution [57,62,63]. Eberhardt [64] proposed that the negative effects of increasing density on population growth are greatest in juvenile survival, followed in turn by the onset of puberty, fecundity and, finally, adult survival. In large mammals, density dependence is most commonly identified in vital rates that influence recruitment, in particular, juvenile survival, and less frequently in adult survival [4,12,19,65]. Indeed, there was evidence for density-dependent regulation in a small elephant seal population at Marion Island operating through changes to fertility [46], and it has also been shown that elephant seal population growth is highly sensitive to adult fertility [22]. However, Pistorius & Bester [66] dismissed juvenile survival as an important driver of change in population growth at Marion Island. This may be explained by the relatively small population at Marion and direct evidence that a decline in the age of female primiparity has occurred there recently [46], suggesting that the dominant mechanisms driving the phenomenology of self limitation in the Marion and Macquarie Island populations may be different.

Our results have important implications for the assessment of environmental change in the Southern Ocean and Antarctic region. Given their status as upper trophic-level predators foraging over vast areas of the subantarctic and Antarctic oceanic zone, variation in abundance and life history parameters in this species may be indicative of larger changes occurring throughout the Antarctic ecosystem [21]. The extensive demographic and population abundance data for the Macquarie Island population now span approximately five elephant seal generations [22], so these datasets consequently represent an invaluable source of information to determine long-term trends in this region. Elephant seal populations throughout the Southern Ocean have declined substantially over the last 50 years, although some populations are demonstrating recent stability or even recovery [22]. Our results highlight the sensitivity of the species to long-term environmental fluctuations and argue for continued monitoring to determine the extent to which deterministic or oscillatory dynamics are affecting the region's higher predator guild.

## Conclusion

Our study quantified the degree to which the likely drivers of variation in abundance interact in a single population of a wide-ranging oceanic predator. The population and demographic models that were constructed provided a quantitative assessment of the complex interactions of extrinsic and intrinsic factors regulating the population, and the processes described provide a comprehensive overview of large mammal dynamics when they are exposed to highly variable environments. Understanding the complex nexus that emerges between these two major forces is a vital precursor for predictions of the influence of rapid climate change on animal populations worldwide. As such, species amenable to long-term monitoring within highly stochastic environments such as the Southern Ocean can act as climatic 'canaries' that chronicle catastrophic ecosystem degradation resulting from human-mediated climate change.

## Methods

### Marking and resighting

Capture-mark-recapture (CMR) datasets were collected from the elephant seal populations breeding on the northern isthmus of Macquarie Island (54°30' S, 158°50' E) over two periods of different population density: (1) the high-density era between 1951 and 1965 when the number of breeding females (*n*_*f*_) breeding there was approximately 5000 and (2) the low-density era between 1993 and 1999 when *n*_*f *_was ca. 2500 to 3000 [22,30,31,67]. During both periods of investigation, newly weaned pups were hot-iron branded after weaning in November [for details see [30,68-70]]. During the high-density era, 6506 seals were branded from thirteen cohorts: 1951 to 1965 (excluding 1956 and 1958), with a mean of 500 seals branded each year. During the low-density era, 10721 pups were branded from five cohorts (1993 to 1997, with a mean of 2144 pups marked each year). We have determined previously that branding in this manner has had no long-term effects on the condition or survival of the seals [69,71], and that branding is an appropriate conservation tool [71]. This study was approved by the Antarctic Animal Care and Ionising Radiation Usage Ethics Committee (Department of the Environment, Commonwealth of Australia), and the Tasmanian Parks and Wildlife Service.

Systematic re-sighting searches were made of all the isthmus beaches where most of the marked seals return [30,68]. There was high variation in the frequency and intensity of the searches during the high-density era [26]; the low-density study had a more rigorous re-sighting strategy [22]. On both occasions, reports of marked seals found away from Macquarie Island were rare [22,26,72]. The years with poor search effort from 1961–1985 (see Fig. 1A) (i.e., when recapture rates were consistently low and inestimable in the CMR models described below) were excluded from the analysis.

### Phenomenological evidence for density dependence

We compiled survey data on the relative abundance of breeding elephant seals at Macquarie Island over time for the two periods of investigation (high- and low-density eras) (see [67], [73] and [22] for data and full methods) (Fig 1A). The survey data consisted of complete counts of the entire adult female population done on a single day each year (15 October). This is the standard method to estimate relative population size for southern elephant seals [74].

To determine the strength of evidence for density dependence using phenomenological (abundance) data, we applied the technique of Brook and Bradshaw [24] to each abundance time series. We adopted a multiple-working hypotheses approach based on information-theoretic model selection and multi-model inference [75]. We first defined an *a priori *model set of five population dynamical models [17] used to describe phenomenological time-series data based on variants of the generalized *θ*-logistic population growth model:

log⁡(Nt+1Nt)=r=rm[1−(NtK)θ]+εt
 MathType@MTEF@5@5@+=feaafiart1ev1aaatCvAUfKttLearuWrP9MDH5MBPbIqV92AaeXatLxBI9gBaebbnrfifHhDYfgasaacH8akY=wiFfYdH8Gipec8Eeeu0xXdbba9frFj0=OqFfea0dXdd9vqai=hGuQ8kuc9pgc9s8qqaq=dirpe0xb9q8qiLsFr0=vr0=vr0dc8meaabaqaciaacaGaaeqabaqabeGadaaakeaacyGGSbaBcqGGVbWBcqGGNbWzdaqadaqaamaalaaabaGaemOta40aaSbaaSqaaiabdsha0jabgUcaRiabigdaXaqabaaakeaacqWGobGtdaWgaaWcbaGaemiDaqhabeaaaaaakiaawIcacaGLPaaacqGH9aqpcqWGYbGCcqGH9aqpcqWGYbGCdaWgaaWcbaGaeeyBa0gabeaakmaadmaabaGaeGymaeJaeyOeI0YaaeWaaeaadaWcaaqaaiabd6eaonaaBaaaleaacqWG0baDaeqaaaGcbaGaem4saSeaaaGaayjkaiaawMcaamaaCaaaleqabaacciGae8hUdehaaaGccaGLBbGaayzxaaGaey4kaSIae8xTdu2aaSbaaSqaaiabdsha0bqabaaaaa@4FB2@

where *N*_*t *_= population size at time *t*, *r *= realized population growth rate, *r*_m _= maximal intrinsic population growth rate, *K *= carrying capacity, *θ *permits a nonlinear relationship between rate of increase and abundance. The term *ε*_*t *_has a mean of zero and a variance (*σ*^2^) that reflects environmental variability in *r*. For each high-density and low-density time series we used maximum-likelihood estimation to fit model parameters (via linear regression for the density-independent random walk [RW] and exponential [EX] models, and for the density-dependent Ricker-logistic [RL] and Gompertz-logistic [GL] models; non-linear regression based on Newton optimization was used to fit the density-dependent full *θ*-logistic [TL] model – [76]). An index of Kullback-Leibler information loss, Akaike's Information Criterion corrected for small sample sizes (AIC_*c*_) weights, was used to assign relative strengths of evidence to each model [75]. The relative support for density dependence is simply the summed weights of the three density-dependent models (RL, GL and TL). More details are given in Brook and Bradshaw [24].

### Capture-mark-recapture analysis

Capture history matrices were constructed from the re-sighting histories of individual seals, with multiple re-sights within a year treated as a single sighting. Capture matrices were analyzed using the capture-mark-recapture (CMR) program MARK [77] which provides maximum-likelihood estimates of apparent survival and re-sight probability based on the Cormack-Jolly-Seber (CJS) time-variant model structure and several models appearing as special cases of this general model [11]. The two fundamental parameters estimated in these models are *ϕ*, the apparent survival probability (true survival confounded with permanent emigration – the latter is considered to be low given the high return rate of seals to the relatively isolated Macquarie Island) of individuals between the *n*^th ^and (*n *+ 1)^th ^year (*n *= 1,..., *k *- 1), and *p*, the re-sight probability for all individuals in the *n*^th ^year (*n *= 1,..., *k*) [77].

We tested whether the CJS-model assumptions were met with parametric goodness-of-fit (GOF) tests implemented by the simulation procedures available in MARK [11]. Here, encounter histories are simulated that exactly meet the CJS assumptions by a bootstrap procedure, and then the simulated data are compared to the observed data to test for goodness-of-fit [77]. The variance inflation (over-dispersion) factor, *ĉ*, was calculated from this procedure and used to correct AIC_*c *_values [11]. Different models combining the main parameters and their hypothesized effects (see below) were compared using AIC_*c *_[75,78]. We accounted for some of the potential over-dispersion by using the second-order approximation AIC_*c*_, denoted QAIC_*c *_[75]. Models containing covariates were compared to the general model (time- and age-variant survival) using the information-theoretic evidence ratio (*ER*) [75]. The evidence ratio is calculated as the QAIC_*c *_weight of any one model divided by a simpler comparison model QAIC_*c *_weight. The *ER *therefore estimates how many more times likely the model in question is over the model(s) to which it is being compared [75].

### Sex and age effects

We examined if there were any differences in survival probability between the sexes; there was little evidence for a difference (see Results), so the sexes were pooled. First-year survival is a good indicator of potential recruitment given that naïve elephant seals have the highest risks of dying compared to other age classes [22]. Therefore, we split the datasets into two age classes: first-year (1 year) and "adult" (> 1 year). This allowed for a direct comparison of the effects of the covariates on first-year survival and subsequent population recruitment.

### Environmental conditions

We used annual averages of the Southern Oscillation Index (SOI) [79] to examine the hypothesis that environmental stochasticity affects annual survival probability [22]. The SOI is a measure of El Niño-Southern Oscillation (ENSO), and it reflects the patterns of variability in the weather and sea surface temperatures of the Southern Ocean [80]. We standardized the mean January-October SOI values by subtracting the mean SOI for January to October from a 50-year mean (1950–2000). This period (Jan-Oct) corresponds to the seals' annual winter foraging trips to sea [33,34]. It has been shown that both the average SOI during the newly weaned seals' first foraging trip and the conditions prevailing during the mother's pre-partum foraging (as inferred from weaning mass) both affect first-year survival [22,38,49]. As such, we included measures of the SOI anomaly during both periods as covariates in our *a priori *model sets to examine their relative support (weaning mass data for the entire dataset were unavailable). Two separate CJS model sets were constructed using these two expressions of the SOI conditions: (1) the first set employed the SOI values relating to the first-year seals' first foraging trip (e.g., seals branded in 1993 foraged for the first time in late-1993 and throughout 1994); (2) the second set used the SOI corresponding to the environmental conditions prevailing during the mother's pre-partum foraging (e.g., mothers of seals born in 1993 were foraging over-winter in 1993).

### Population density

Published counts of female seals on the isthmus during the breeding season were used to measure the effect of population density on survival [73]. Both the density of breeding females from the current year, and the density of the breeding females from the previous year were included in the models considered as density covariates. This approach allowed us to examine the possibility of a lag effect of density on survival. The density covariates were standardized by calculating the minimum value in the covariate vector and subtracting this from all other values. The maximum value was calculated for this new vector, and the new vector was then divided by the maximum. The density covariates were incorporated into models with and without the SOI covariates. MARK includes covariates in the CJS model by expressing the natural logarithm of the probability of survival as a logistic function of the covariates:

logit (ϕ)=y−intercept+β(x)(x−x¯SDx)−β(x2)−(x2−x¯2SDx)
 MathType@MTEF@5@5@+=feaafiart1ev1aaatCvAUfKttLearuWrP9MDH5MBPbIqV92AaeXatLxBI9gBaebbnrfifHhDYfgasaacH8akY=wiFfYdH8Gipec8Eeeu0xXdbba9frFj0=OqFfea0dXdd9vqai=hGuQ8kuc9pgc9s8qqaq=dirpe0xb9q8qiLsFr0=vr0=vr0dc8meaabaqaciaacaGaaeqabaqabeGadaaakeaacqqGSbaBcqqGVbWBcqqGNbWzcqqGPbqAcqqG0baDcqqGGaaicqGGOaakiiGacqWFgpGzcqGGPaqkcqGH9aqpcqqG5bqEcqGHsislcqqGPbqAcqqGUbGBcqqG0baDcqqGLbqzcqqGYbGCcqqGJbWycqqGLbqzcqqGWbaCcqqG0baDcqGHRaWkcqWFYoGydaWgaaWcbaGaeiikaGIaemiEaGNaeiykaKcabeaakmaabmaabaWaaSaaaeaacqWG4baEcqGHsislcuWG4baEgaqeaaqaaiabdofatjabdseaejabdIha4baaaiaawIcacaGLPaaacqGHsislcqWFYoGydaWgaaWcbaGaeiikaGIaemiEaG3aaWbaaWqabeaacqaIYaGmaaWccqGGPaqkaeqaaOGaeyOeI0YaaeWaaeaadaWcaaqaaiabdIha4naaCaaaleqabaGaeGOmaidaaOGaeyOeI0IafmiEaGNbaebadaahaaWcbeqaaiabikdaYaaaaOqaaiabdofatjabdseaejabdIha4baaaiaawIcacaGLPaaaaaa@6A52@

where logit(*ϕ*) is the logit-transformed survival estimate of a seal with the covariate *x*, *β *is the logit function calculated in MARK for *x *and SD is the standard deviation of *x*. This function is fixed in the log-likelihood for survival as in a logistic regression. This model assumes that there is an optimal value of the covariate and that there are some selective penalties associated with extreme values [77].

## Authors' contributions

SCD took the lead in writing the manuscript and did the analyses. CJAB, MAH and CRM initiated the study, and CJAB contributed to data analyses. All authors assisted in writing the manuscript and approved the final version.

## Supplementary Material

Additional file 1 Additional tables showing the capture-mark-recapture model rankings using increasing values of over-dispersion. The additional tables demonstrate the change in information-theoretic (*w*QAIC_*c*_) ranking of models examining the effects of density, age, sex, time and the Southern Oscillation Index (SOI) on apparent survival (*ϕ*) and recapture probability (*p*) of southern elephant seals (*Mirounga leonina*) at Macquarie Island with increasing values of over-dispersion (*ĉ*). Click here for file
