# Supplementary material for: Complex interplay between intrinsic and extrinsic drivers of long-term survival trends in southern elephant seals
Source: BMC Ecol. 2007 Mar 27;7:3. doi: 10.1186/1472-6785-7-3 (PMC1855316; doi:10.1186/1472-6785-7-3)
Supplement: Additional file 1 — Additional tables showing the capture-mark-recapture model rankings using increasing values of over-dispersion. The additional tables demonstrate the change in information-theoretic (wQAICc) ranking of models examining the effects of density, age, sex, time and the Southern Oscillation Index (SOI) on apparent survival (ϕ) and recapture probability (p) of southern elephant seals (Mirounga leonina) at Macquarie Island with increasing values of over-dispersion (ĉ). [file 1472-6785-7-3-S1.doc]

# Additional files

# Additional file 1

## Additional tables showing the capture-mark-recapture model rankings using increasing values of over-dispersion.

The following tables demonstrate the change in information-theoretic (*w*QAIC*_c_*) ranking of models examining the effects of density, age, sex, time and the Southern Oscillation Index (SOI) on apparent survival (*φ*) and recapture probability (*p*) of southern elephant seals (*Mirounga leonina*) at Macquarie Island with increasing values of over-dispersion ($\hat{c}$).

## Additional Table 1 – Model ranking for models estimating age-specific survival and recapture probability with increasing over-dispersion

|  |  | ***w*QAIC*_c_*** | | | | | | | | | |
| --- | --- | --- | --- | --- | --- | --- | --- | --- | --- | --- | --- |
| **Model** | ***k*** | $\hat{c}$**=1** | $\hat{c}$**=2** | $\hat{c}$**=3** | $\hat{c}$**=4** | $\hat{c}$**=5** | $\hat{c}$**=6** | $\hat{c}$**=7** | $\hat{c}$**=8** | $\hat{c}$**=9** | $\hat{c}$**=10** |
| (A) |  |  |  |  |  |  |  |  |  |  |  |
| *φ*(**age**-**t**/**t**) *p*(**age**-**t**/**t**) | 17 | **1.000** | **0.996** | **0.909** | **0.673** | 0.444 | 0.297 | 0.209 | 0.152 | 0.111 | 0.079 |
| *φ*(**age**-**t**/**t**) *p*(**t**) | 13 | <0.001 | 0.004 | 0.091 | 0.327 | **0.555** | **0.698** | **0.771** | **0.786** | **0.743** | **0.651** |
| *φ*(**t**) *p*(**age**-**t**/**t**) | 13 | <0.001 | <0.001 | <0.001 | <0.001 | <0.001 | 0.002 | 0.004 | 0.009 | 0.013 | 0.018 |
| *φ*(**t**) *p*(**t**) | 9 | <0.001 | <0.001 | <0.001 | <0.001 | <0.001 | 0.001 | 0.004 | 0.012 | 0.029 | 0.053 |
|  |  |  |  |  |  |  |  |  |  |  |  |
| (B) |  |  |  |  |  |  |  |  |  |  |  |
| *φ*(**age**-**t**/**t**) *p*(**t**) | 13 | **0.830** | **0.958** | **0.969** | **0.973** | **0.975** | **0.976** | **0.977** | **0.977** | **0.974** | **0.966** |
| *φ*(**age**-**sex*****t**/**sex*****t**) *p*(**t**) | 17 | 0.098 | 0.004 | 0.001 | 0.001 | <0.001 | <0.001 | <0.001 | <0.001 | <0.001 | <0.001 |
| *φ*(**age**-**sex*****t**/**t**) *p*(**t**) | 22 | 0.073 | 0.038 | 0.030 | 0.026 | 0.024 | 0.023 | 0.022 | 0.022 | 0.021 | 0.021 |
| *φ*(**t*****sex**) *p*(**t**) | 9 | <0.001 | <0.001 | <0.001 | <0.001 | <0.001 | <0.001 | <0.001 | <0.001 | <0.001 | <0.001 |
| *φ*(**t**) *p*(**t**) | 8 | <0.001 | <0.001 | <0.001 | <0.001 | <0.001 | <0.001 | <0.001 | 0.001 | 0.002 | 0.006 |
| *φ*(**t*****sex**) *p*(**t*****sex**) | 14 | <0.001 | <0.001 | <0.001 | <0.001 | <0.001 | <0.001 | <0.001 | <0.001 | <0.001 | <0.001 |
| *φ*(**t**) *p*(**t*****sex**) | 14 | <0.001 | <0.001 | <0.001 | <0.001 | <0.001 | <0.001 | <0.001 | <0.001 | <0.001 | <0.001 |
| *φ*(**sex**) *p*(**t**) | 18 | <0.001 | <0.001 | <0.001 | <0.001 | <0.001 | <0.001 | <0.001 | 0.001 | 0.002 | 0.007 |
| *φ*(**sex**) *p*(**t*****sex**) | 14 | <0.001 | <0.001 | <0.001 | <0.001 | <0.001 | <0.001 | <0.001 | <0.001 | <0.001 | <0.001 |
| *φ*(**t**) *p*(**sex**) | 8 | <0.001 | <0.001 | <0.001 | <0.001 | <0.001 | <0.001 | <0.001 | <0.001 | <0.001 | <0.001 |
| *φ*(**t*****sex**) *p*(**sex**) | 14 | <0.001 | <0.001 | <0.001 | <0.001 | <0.001 | <0.001 | <0.001 | <0.001 | <0.001 | <0.001 |
| *φ*(**sex**) *p*(**sex**) | 4 | <0.001 | <0.001 | <0.001 | <0.001 | <0.001 | <0.001 | <0.001 | <0.001 | <0.001 | <0.001 |

(A) Effects of time (**t**) and age (**age**–**juvenile/adult**) and (B) time (**t**), age (**age**–**juvenile/adult**) and sex (**sex**) on the probability of survival (*φ*) and recapture (*p*) of southern elephant seals during the low-density era (1993-1999). Shown is the change in model ranking as the inflation factor ($\hat{c}$) increases incrementally from 1 to 10. Models are ranked according to their Akaike weights (*w*QAIC*_c_*). Also shown are the number of parameters (*k*). Highest-ranking model weights for each value of $\hat{c}$ are shown in boldface.

## Additional Table 2 – Model ranking for models estimating age-specific survival and recapture probability as a function of environmental stochasticity and population density with increasing over-dispersion

|  |  | ***w*QAIC*_c_*** | | | | | | | | | |
| --- | --- | --- | --- | --- | --- | --- | --- | --- | --- | --- | --- |
| **Model** | ***k*** | $\hat{c}$**=1** | $\hat{c}$**=2** | $\hat{c}$**=3** | $\hat{c}$**=4** | $\hat{c}$**=5** | $\hat{c}$**=6** | $\hat{c}$**=7** | $\hat{c}$**=8** | $\hat{c}$**=9** | $\hat{c}$**=10** |
| (A) |  |  |  |  |  |  |  |  |  |  |  |
| *φ*(**age**-**mother*****t**/**mother*****t**) *p*(**t**) | 24 | **0.982** | 0.265 | 0.064 | 0.028 | 0.017 | 0.012 | 0.009 | 0.007 | 0.006 | 0.004 |
| *φ*(**mother*****t**) *p*(**t**) | 18 | 0.019 | **0.734** | **0.931** | **0.956** | **0.945** | **0.915** | **0.872** | **0.817** | **0.746** | **0.655** |
| *φ*(**age**-**pup*****t**/**pup*****t**) *p*(**t**) | 22 | <0.001 | 0.001 | 0.004 | 0.005 | 0.007 | 0.008 | 0.008 | 0.008 | 0.008 | 0.008 |
| *φ*(**age**-**t**/**t**) *p*(**t**) | 17 | <0.001 | <0.001 | 0.001 | 0.009 | 0.029 | 0.059 | 0.096 | 0.134 | 0.167 | 0.189 |
| *φ*(**age**-**pup*****t**/**t**) *p*(**t**) | 21 | <0.001 | <0.001 | <0.001 | <0.001 | 0.001 | 0.001 | 0.002 | 0.002 | 0.003 | 0.003 |
| *φ*(**age**-**mother*****t**/**t**) *p*(**t**) | 23 | <0.001 | <0.001 | <0.001 | <0.001 | <0.001 | <0.001 | <0.001 | <0.001 | <0.001 | <0.001 |
| *φ*(**pup*****t**) *p*(**t**) | 18 | <0.001 | <0.001 | <0.001 | <0.001 | 0.002 | 0.004 | 0.009 | 0.015 | 0.021 | 0.027 |
| *φ*(**t**)*p*(**t**) | 11 | <0.001 | <0.001 | <0.001 | <0.001 | <0.001 | <0.001 | 0.004 | 0.016 | 0.049 | 0.113 |
|  |  |  |  |  |  |  |  |  |  |  |  |
| (B) |  |  |  |  |  |  |  |  |  |  |  |
| *φ*(**age**-**t**/**t**) *p*(**t**) | 13 | **0.754** | 0.200 | 0.094 | 0.062 | 0.047 | 0.039 | 0.034 | 0.031 | 0.028 | 0.026 |
| *φ*(**age**-**density**/**t**) *p*(**t**) | 10 | 0.165 | **0.420** | **0.420** | **0.403** | **0.387** | **0.373** | **0.362** | **0.352** | **0.343** | **0.334** |
| *φ*(**age**-**dlag**/**t**) *p*(**t**) | 10 | 0.072 | 0.278 | 0.319 | 0.328 | 0.328 | 0.325 | 0.322 | 0.317 | 0.313 | 0.308 |
| *φ*(**age**-**density**/**density**) *p*(**t**) | 10 | 0.010 | 0.102 | 0.164 | 0.198 | 0.220 | 0.233 | 0.241 | 0.247 | 0.250 | 0.252 |
| *φ*(**age**-**dlag**/**dlag**) *p*(**t**) | 10 | <0.001 | <0.001 | 0.003 | 0.009 | 0.018 | 0.029 | 0.040 | 0.052 | 0.062 | 0.072 |
| *φ*(**t**) *p*(**t**) | 9 | <0.001 | <0.001 | <0.001 | <0.001 | <0.001 | <0.001 | <0.001 | <0.001 | 0.001 | 0.002 |
| *φ*(**dlag**) *p*(**t**) | 8 | <0.001 | <0.001 | <0.001 | <0.001 | <0.001 | <0.001 | <0.001 | 0.001 | 0.002 | 0.003 |
| *φ*(**density**) *p*(**t**) | 8 | <0.001 | <0.001 | <0.001 | <0.001 | <0.001 | <0.001 | <0.001 | 0.001 | 0.001 | 0.003 |

(A) Effects of age (**age**–**juvenile/adult**), time (**t**) and environmental conditions (SOI during a newly weaned seal’s foraging period [**pup**] and during a mother’s pre-partum foraging period [**mother**] and (B) age (**age**–**juvenile/adult**), time (**t**), density of breeding females (**density**) and density of breeding females lagged by one year (**dlag**) on the probability of survival (*φ*) in southern elephant seals during the low-density era (1993-1998). Shown is the change in model ranking as the inflation factor ($\hat{c}$) increases incrementally from 1 to 10. Models are ranked according to their Akaike weights (*w*QAIC*_c_*). Also shown are the number of parameters (*k*). Highest-ranking model weights for each value of $\hat{c}$ are shown in boldface.

## Additional Table 3 – Model ranking for models estimating age-specific survival and recapture probability as a function of environmental stochasticity and population density with increasing over-dispersion

|  |  | ***w*QAIC*_c_*** | | | | | | | | | |
| --- | --- | --- | --- | --- | --- | --- | --- | --- | --- | --- | --- |
| **Model** | ***k*** | $\hat{c}$**=1** | $\hat{c}$**=2** | $\hat{c}$**=3** | $\hat{c}$**=4** | $\hat{c}$**=5** | $\hat{c}$**=6** | $\hat{c}$**=7** | $\hat{c}$**=8** | $\hat{c}$**=9** | $\hat{c}$**=10** |
|  |  |  |  |  |  |  |  |  |  |  |  |
| *φ*(**age**-**mother*****t**/**mother*****t**) *p*(**t**) | 22 | **0.721** | **0.415** | 0.113 | 0.021 | 0.005 | 0.002 | 0.001 | <0.001 | <0.001 | <0.001 |
| *φ*(**age**-**t**/**mother*****t**) *p*(**t**) | 23 | 0.274 | 0.155 | 0.042 | 0.008 | 0.002 | 0.001 | <0.001 | <0.001 | <0.001 | <0.001 |
| *φ*(**mother*****t**) *p*(**t**) | 17 | 0.005 | 0.423 | **0.607** | 0.264 | 0.097 | 0.044 | 0.024 | 0.014 | 0.010 | 0.007 |
| *φ*(**age**-**pup*****t**/**pup*****t**) *p*(**t**) | 20 | <0.001 | 0.002 | 0.006 | 0.004 | 0.002 | 0.001 | 0.001 | <0.001 | <0.001 | <0.001 |
| *φ*(**age**-**t**/**pup*****t**) *p*(**t**) | 25 | <0.001 | <0.001 | <0.001 | <0.001 | <0.001 | <0.001 | <0.001 | <0.001 | <0.001 | <0.001 |
| *φ*(**age**-**density**+**mother**/**density**+**mother**) *p*(**t**) | 12 | <0.001 | 0.004 | 0.144 | **0.315** | **0.305** | 0.260 | 0.222 | 0.193 | 0.170 | 0.151 |
| *φ*(**age**-**density**+**pup**/**density**+**pup**) *p*(**t**) | 12 | <0.001 | <0.001 | 0.021 | 0.073 | 0.095 | 0.099 | 0.097 | 0.093 | 0.089 | 0.084 |
| *φ*(**age**-**density**+**mother**/**t**) *p*(**t**) | 14 | <0.001 | <0.001 | 0.004 | 0.014 | 0.017 | 0.016 | 0.016 | 0.015 | 0.014 | 0.013 |
| *φ*(**age**-**density**+**mother**/**density**+**mother**) *p*(**t**) | 12 | <0.001 | <0.001 | 0.016 | 0.061 | 0.082 | 0.087 | 0.087 | 0.085 | 0.081 | 0.078 |
| *φ*(**age**-**t**/**t**) *p*(**t**) | 17 | <0.001 | <0.001 | <0.001 | 0.001 | 0.001 | 0.001 | 0.001 | 0.001 | 0.001 | 0.001 |
| *φ*(**age**-**density**/**t**) *p*(**t**) | 12 | <0.001 | <0.001 | 0.009 | 0.038 | 0.056 | 0.063 | 0.066 | 0.067 | 0.066 | 0.064 |
| *φ*(**age**-**density**/**density**) *p*(**t**) | 10 | <0.001 | <0.001 | 0.032 | 0.169 | 0.276 | **0.335** | **0.367** | **0.384** | **0.391** | **0.390** |
| *φ*(**age**-**dlag**+**mother**/**t**) *p*(**t**) | 16 | <0.001 | <0.001 | 0.001 | 0.002 | 0.002 | 0.002 | 0.002 | 0.002 | 0.002 | 0.002 |
| *φ*(**age**-**dlag**+**pup**/**t**) *p*(**t**) | 16 | <0.001 | <0.001 | <0.001 | 0.001 | 0.002 | 0.002 | 0.002 | 0.002 | 0.002 | 0.002 |
| *φ*(**age**-**density**/**t**) *p*(**t**) | 14 | <0.001 | <0.001 | 0.002 | 0.006 | 0.009 | 0.010 | 0.010 | 0.010 | 0.010 | 0.009 |
| *φ*(**age**-**density**+**pup**/**t**) *p*(**t**) | 16 | <0.001 | <0.001 | <0.001 | 0.001 | 0.001 | 0.002 | 0.002 | 0.002 | 0.001 | 0.001 |
| *φ*(**age**-**pup*****t**/**t**) *p*(**t**) | 20 | <0.001 | <0.001 | <0.001 | <0.001 | <0.001 | <0.001 | <0.001 | <0.001 | <0.001 | <0.001 |
| *φ*(**age**-**dlag**+**pup**/**dlag**+**pup**) *p*(**t**) | 12 | <0.001 | <0.001 | 0.002 | 0.014 | 0.026 | 0.033 | 0.038 | 0.041 | 0.043 | 0.044 |
| *φ*(**age**-**mother*****t**/**t**) *p*(**t**) | 22 | <0.001 | <0.001 | <0.001 | <0.001 | <0.001 | <0.001 | <0.001 | <0.001 | <0.001 | <0.001 |
| *φ*(**pup*****t**) *p*(**t**) | 17 | <0.001 | <0.001 | <0.001 | <0.001 | <0.001 | <0.001 | <0.001 | <0.001 | <0.001 | <0.001 |
| *φ*(**age**-**dlag**/**dlag**) *p*(**t**) | 10 | <0.001 | <0.001 | 0.001 | 0.007 | 0.023 | 0.042 | 0.061 | 0.080 | 0.097 | 0.112 |
| *φ*(**density**+**pup**) *p*(**t**) | 9 | <0.001 | <0.001 | <0.001 | <0.001 | <0.001 | <0.001 | 0.002 | 0.004 | 0.007 | 0.011 |
| *φ*(**dlag**+**pup**) *p*(**t**) | 9 | <0.001 | <0.001 | <0.001 | <0.001 | <0.001 | <0.001 | 0.002 | 0.004 | 0.007 | 0.011 |
| *φ*(**density**+**mother**) *p*(**t**) | 9 | <0.001 | <0.001 | <0.001 | <0.001 | <0.001 | <0.001 | <0.001 | 0.001 | 0.002 | 0.004 |
| *φ*(**dlag**+**mother**) *p*(**t**) | 9 | <0.001 | <0.001 | <0.001 | <0.001 | <0.001 | <0.001 | <0.001 | 0.001 | 0.002 | 0.004 |
| *φ*(**t**) *p*(**t**) | 10 | <0.001 | <0.001 | <0.001 | <0.001 | <0.001 | <0.001 | <0.001 | <0.001 | 0.001 | 0.001 |
| *φ*(**dlag**) *p*(**t**) | 8 | <0.001 | <0.001 | <0.001 | <0.001 | <0.001 | <0.001 | <0.001 | 0.001 | 0.003 | 0.005 |
| *φ*(**density**) *p*(**t**) | 8 | <0.001 | <0.001 | <0.001 | <0.001 | <0.001 | <0.001 | <0.001 | 0.001 | 0.002 | 0.005 |

Effects of age (**age**–**juvenile/adult**), time (**t**), density of breeding females (**density**), density of breeding females lagged by one year (**dlag**) and environmental conditions (SOI during a newly weaned seal’s foraging period [**pup**] and during a mother’s pre-partum foraging period [**mother**] on the probability of survival (*φ*) in southern elephant seals during the low-density era (1993-1998). Shown is change in model ranking as the inflation factor ($\hat{c}$) increases incrementally from 1 to 10. Models are ranked according to their Akaike weights (*w*QAIC*_c_*). Also shown are number of parameters (*k*). Highest-ranking model weights for each value of $\hat{c}$ are shown in boldface.
